# Mannose Receptor (MR) Engagement by Mesothelin GPI Anchor Polarizes Tumor-Associated Macrophages and Is Blocked by Anti-MR Human Recombinant Antibody

**DOI:** 10.1371/journal.pone.0028386

**Published:** 2011-12-06

**Authors:** Denarda Dangaj, Karen L. Abbott, Ananda Mookerjee, Aizhi Zhao, Pamela S. Kirby, Raphael Sandaltzopoulos, Daniel J. Powell, Antonin Lamazière, Don L. Siegel, Claude Wolf, Nathalie Scholler

**Affiliations:** 1 Department of Obstetrics and Gynecology, Penn Ovarian Cancer Research Center, University of Pennsylvania, Philadelphia, Pennsylvania, United States of America; 2 Department of Molecular Biology and Genetics, Democritus University of Thrace, Alexandroupolis, Greece; 3 Complex Carbohydrate Research Center, University of Georgia, Athens, Georgia, United States of America; 4 Department of Pathology and Laboratory Medicine, University of Pennsylvania, Philadelphia, Pennsylvania, United States of America; 5 Department of Biochemistry, School of Medicine Saint Antoine, Université Pierre et Marie Curie, Paris, France; Institute of Molecular and Cell Biology, Singapore

## Abstract

Tumor-infiltrating macrophages respond to microenvironmental signals by developing a tumor-associated phenotype characterized by high expression of mannose receptor (MR, CD206). Antibody cross-linking of CD206 triggers anergy in dendritic cells and CD206 engagement by tumoral mucins activates an immune suppressive phenotype in tumor-associated macrophages (TAMs). Many tumor antigens are heavily glycosylated, such as tumoral mucins, and/or attached to tumor cells by mannose residue-containing glycolipids (GPI anchors), as for example mesothelin and the family of carcinoembryonic antigen (CEA). However, the binding to mannose receptor of soluble tumor antigen GPI anchors via mannose residues has not been systematically studied. To address this question, we analyzed the binding of tumor-released mesothelin to ascites-infiltrating macrophages from ovarian cancer patients. We also modeled functional interactions between macrophages and soluble mesothelin using an *in vitro* system of co-culture in transwells of healthy donor macrophages with human ovarian cancer cell lines. We found that soluble mesothelin bound to human macrophages and that the binding depended on the presence of GPI anchor and of mannose receptor. We next challenged the system with antibodies directed against the mannose receptor domain 4 (CDR4-MR). We isolated three novel anti-CDR4-MR human recombinant antibodies (scFv) using a yeast-display library of human scFv. Anti-CDR4-MR scFv #G11 could block mesothelin binding to macrophages and prevent tumor-induced phenotype polarization of CD206^low^ macrophages towards TAMs. Our findings indicate that tumor-released mesothelin is linked to GPI anchor, engages macrophage mannose receptor, and contributes to macrophage polarization towards TAMs. We propose that compounds able to block tumor antigen GPI anchor/CD206 interactions, such as our novel anti-CRD4-MR scFv, could prevent tumor-induced TAM polarization and have therapeutic potential against ovarian cancer, through polarization control of tumor-infiltrating innate immune cells.

## Introduction

Macrophages show a remarkable degree of plasticity and exert diverse functions, depending on the microenvironmental stimuli [1]. Macrophages activated toward a classical, proinflammatory phenotype (M1) elicit anti-tumor activity and promote TH1 immune responses [2], while macrophages with an alternative phenotype (M2) promote TH2 immune responses and tissue remodelling. Tumor polarization of macrophages represents an essential immune escape mechanism that results in a hampered innate immune response leading to a poor adaptive immunity [3], [4]. Recent studies suggest that tumor-induced differentiation of macrophages is a continuous process with several intermediate phenotypic states [5], [6], possibly reversible [7]. Tumor-associated macrophages (TAMs) share properties with M2 macrophages, including high expression of IL10 and mannose receptor (CD206), and low expression of IL-12 [8]. TAMs constitute a predominant cell population of the tumor microenvironment and are correlated with poor clinical outcome [9]. However, the identification of factors responsible for TAM polarization is not complete. Mouse studies suggest a critical role for CSF-1 in attracting monocytes at the tumor site [1], while cytokine imbalance in favour of IL-10 and TGF-β in the microenvironment could foster immunosuppression and polarize macrophages to elicit pro-tumoral functions [10]. Hagemann and colleagues have also proposed that macrophage differentiation towards TAMs involves a “chemical conversation” via exchange of soluble extracellular mediators between ovarian tumor cells and macrophages [11], [12].

TAMs abundantly express mannose receptor (MR/CD206) [13], [14]. CD206 is a highly conserved calcium-dependent multilectin and a pattern recognition receptor (PRR) that mediates non-opsonic phagocytic uptake of a wide variety of microbes and that also functions as an endocytic receptor for glycans [15], [16], [17], [18]. CD206 comprises of two distinct extracellular lectin binding sites, one that recognizes sulfated sugars [19], [20] and another that preferentially binds to branched sugars with terminal mannose, fucose or N-acetyl-glycosamine [21], [22], [23], [24]. Although the role of CD206 in innate immunity is well described [25], [26], its contribution to tumor immunity remains understudied. Recent evidence demonstrated that CD206 promotes the circulation of lymphocytes and tumor cells through the lymphatics and to the draining lymph nodes [27]. In addition, CD206 cross-linking with an anti-MR mAb (clone PAM-1) can drive DCs differentiation into APCs promoting T-cell anergy [28], which contributes to the failure of the immune surveillance against solid tumors and facilitates tumor growth and spreading [29], [30], [31].

Ovarian cancer releases various glycoproteins and many of these tumor antigens have been evaluated as biomarkers [32]. Well-studied tumor antigens such as mesothelin [33], CEA [34], [35], and folate receptor [36], [37] are displayed to the cell surface through a GPI-anchor. GPI anchor proteins are structurally and functionally diverse and play vital roles in numerous biological processes [38], [39], including cell adhesion, localization on a specific membrane, association with other membrane proteins and cell signaling [40]. GPI-anchors are evolutionary conserved and their presence on parasite surface proteins activates PI3K pro-inflammatory pathway upon interaction with host macrophages [41]. GPI-anchored glycoproteins are associated to lipid raft domains [42], [43] that are characterized by a liquid ordered arrangement of lipids depending on highly saturated sphingomyelin species (SM) tightly associated with cholesterol (CHOL). CHOL/SM ratio is typically close to 1 in lipid raft [44], [45], and a high SM/Phosphatidylcholine (PC) ratio thought to maintain low polyunsaturated glycerophospholipids is also characteristic of lipid rafts, as compared with more fluid fractions of the membrane [46]. GPI anchors are released from cell membranes by two main mechanisms, shedding of intact GPI anchors in complexes with membrane lipids or in membrane vesicles (exosomes) [47], and proteolytic cleavage mediated by the bacterial GPI-phospholipase C (GPI-PLC), the mammalian GPI-phospholipase D (GPI-PLD) [48], or by the angiotensin-converting enzyme (ACE) that frees terminal mannose [49].

Mesothelin is a GPI-anchored cancer biomarker over-expressed by lung cancers, mesotheliomas, pancreatic and ovarian adenocarcinomas [33] . It is also a soluble biomarker detectable in body fluids of patients with epithelial cancers [36], [50], [51], [52], [53], [54]. Mesothelin binds with high affinity to CA125 through glycan interaction and mediates heterotypic cell adhesion that may be involved in ovarian carcinoma pathogenesis and micrometastatic disease [55], [56], [57]. However, despite some progress [58], [59], [60], the role of mesothelin during cancer development remains to be fully understood.

We hypothesized that that MR engagement by tumor-released mesothelin contributes to macrophage polarization. We further hypothesized that tumor-released mesothelin binds to mannose receptor expressed by macrophages via GPI anchor-mannose residues. To address these questions, we used soluble mesothelin from patient samples, tumor cell lines and cells transfected with a GPI-truncated form of mesothelin. Binding experiments were performed in medium and in the presence of blocking reagents such as mannan, a high affinity ligand for mannose receptor [28], [61], [62], or of novel recombinant antibodies of human origin (scFv) directed against the mannose receptor domain 4 (CDR4-MR). Alterations of macrophage polarization were monitored by qRT-PCR, flow cytometry, and bead-based arrays. The demonstration of the attachment of a GPI anchor to soluble mesothelin was performed by ELISA assays, tandem mass spectrometry and co-immunoprecipitation.

## Results

### Tumor-released mesothelin binds to ascites-infiltrating macrophages from ovarian cancer patients

**Figure 1:**
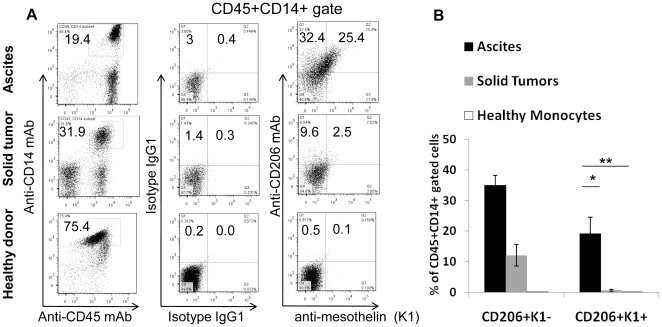
Mesothelin binding to CD14^+^ ascites-infiltrating cells from ovarian cancer patients. **A.** Total cells from ascites (upper panels) or solid tumors (middle panels) from ovarian cancer patients gated on CD14^+^ CD45^+^ (left panels) after exclusion of cells stained with EpCam and 7-AAD. Gated cells were labeled with APC anti-CD206 and PE anti-mesothelin K1, as indicated. CD14^+^ CD45^+^ cells freshly purified from peripheral blood of healthy donors (lower panels) and isotypes IgG1 (APC and PE) were used as negative controls. **B.** Percentages of CD45^+^CD14^+^CD206^+^ cells in solid tumors (**grey bars**, n = 8), ascites samples (**black bars**, n = 6), and healthy donors (**white bars**, n = 12) that bind (CD206^+^ K1^+^) or not (CD206^+^ K1^-^) to mesothelin. Statistical significance was determined by unpaired t-test analysis (***, *P = *0.001). Error bars represent standard error of mean (SEM).

To explore whether tumor-released mesothelin could bind to macrophages, frozen cells isolated from human ascites (n = 6) or from solid tumors (n = 8) of ovarian cancer patients, as well as healthy donor monocytes (n = 12), were stained with anti-Epcam, anti-CD45, anti-CD14, anti-CD206, anti-mesothelin (K1) mAbs and 7-AAD. Viable Epcam^−^CD45^+^CD14^+^ cells were gated and analyzed for CD206 expression and binding to soluble mesothelin. **Figure 1** shows that the majority of CD45^+^CD14^+^ cells from ascites samples expressed high level of CD206 and soluble mesothelin bound to about a fifth of them (**Fig. 1A** **upper panels and** **Fig. 1B**). Lower levels of CD206 were expressed by CD45^+^CD14^+^ cells from solid tumor samples and mesothelin bound only to a low percentage of them (**Fig. 1A** **middle panels and** **Fig. 1B**). Finally, none of the healthy donor CD45^+^CD14^+^ cells expressed CD206 or bound to anti-mesothelin K1 antibody (**Fig. 1A** **lower panels and** **Fig. 1B**). These results were the first evidence that soluble mesothelin could bind to ascites-infiltrating CD206^high^ macrophages from ovarian cancer patients and to some tumor-infiltrating CD206^low^ macrophages.

### Tumor-released mesothelin binds to CD206^high^ monocytes from normal donors

**Figure 2:**
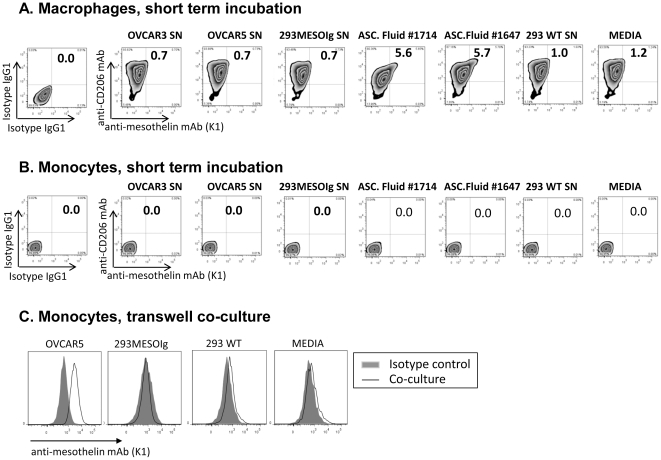
Mesothelin binding to healthy donor monocytes and macrophages co-cultured with tumor cells. **A–B.** Short term incubations. *In vitro* differentiated CD206^+^ macrophages (**A**) and CD206^−^ monocytes (**B**) from healthy donors were incubated with conditioned media from OVCAR3, OVCAR5, 293 MESOIg, or ascites fluids from patients #1714 and #1647 after blocking with 10 µg/ml of human IgG. As negative controls, cells were incubated with conditioned media from 293WT cell line, RPMI+10% FBS medium. Isotype control IgG1 antibodies (APC and PE) on cells incubated with RPMI/FBS. **C.** Transwell co-cultures: Freshly isolated monocytes were cocultured with OVCAR5 or 293mesoIg. As negative controls, monocytes were incubated with 293WT or with RPMI+10% FBS medium only. **Grey area**s, PE isotype IgG1 control; **open area**, PE-conjugated anti-mesothelin mAb (K1). Results representative of 3 or more independent experiments.

To model the binding of mesothelin to macrophages, we set up two types of *in vitro* assay systems using healthy donor monocytes and *in vitro* differentiated CD206^low/high^ macrophages that were 1/ briefly incubated with conditioned media or with ascites fluids, or 2/ co-cultured for 3 days in transwells with OVCAR5 ovarian cancer cell line or with 293 MESOIg secreting GPI-truncated mesothelin [63] and, as controls, with wild type 293 cell line. CD206^low^ and CD206^high^ macrophage phenotypes are illustrated in **Fig. S1.** After 30 min incubation, mesothelin from ascites fluids bound to CD206^high^ macrophages (**Fig. 2A** **,**) but mesothelin from cell line conditioned media did not (**Fig. 2A**). Monocytes were not bound by soluble mesothelin in any of these conditions (**Fig. 2B**). However, after 3 days of transwell co-culture, OVCAR5-released mesothelin bound to monocytes (**Fig. 2C** **,**) but 293 MESOIg-released mesothelin did not (**Fig. 2C**). These results show that mesothelin binding to healthy donor monocytes was proportional to CD206 expression, which supported the hypothesis that soluble mesothelin bound to CD206. In addition, the lack of binding of GPI-truncated mesothelin to monocytes suggested that GPI anchor contributed, at least in part, to mesothelin binding to CD206.

### Tumor-released mesothelin binds to CD206

**Figure 3:**
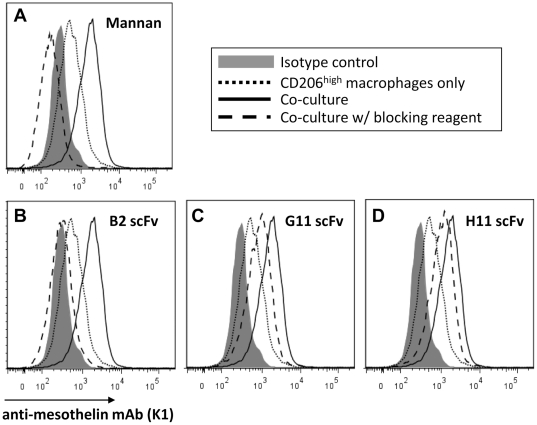
Blocking of mesothelin binding to CD206^high^ macrophages with mannan or anti-CRD4 MR scFvs. Macrophages were labeled with PE anti-mesothelin antibody K1 (**lines**) or with PE isotype control Ab (**grey area**) after *in vitro* differentiation with IL4/IL10 and 72 hr co-culture with OVCAR3 cells (**bold lines**). Blocking conditions (**dashed lines**) included (**A**) mannan, or (**B–D**) anti-CRD4 MR scFvs #B2 (**B**), #G11 (**C**) or #H11 (**D**). As control, CD206^high^ macrophages were incubated in medium only (**dotted lines**). Results representative of 3 or more independent experiments.

To further assess whether mesothelin binding was mediated through mannose receptor, we co-cultured CD206^high^ macrophages and OVCAR3 tumor cells in medium or in the presence of mannan, a high affinity ligand for mannose. **Figure 3A** shows that the presence of 1 mg/ml of mannan during the co-culture completely abrogated the binding of tumor-cell released mesothelin to CD206^high^ macrophages.

To confirm that mesothelin binding occurred specifically through mannose receptor, rather than through other lectins also bound by mannan [64], we sought to isolate recombinant antibodies (scFv) specific for the mannose binding domain of mannose receptor (CRD4-MR) [65]. To do so, we isolated anti-CRD4-MR scFvs from a novel yeast-display human scFv library [66], using a combination of magnetic and flow sorting [63], [66], [67], [68] and a yeast-secreted recombinant CRD4-MR protein (rCRD4-MR).

Three anti-CRD4-MR scFvs (G11, B2 and H11) were isolated and sequenced (**Table S1).** The scFv analysis was performed using the Kabat system from the NCBI Ig blast website (http://www.ncbi.nlm.nih.gov/igblast/). B2 and G11 CDR3s on the nucleotide level look almost the same except for somatic mutation, which implies that they both came from the same B-cell clone**.** Anti-CRD4-MR scFvs were validated for binding to rCRD4-MR by ELISA (**Fig. S2A**), and to CD206^low^ and CD206^high^ macrophages by flow cytometry (**Fig. S2B**). While anti-CRD4-MR #G11 and #B2 scFvs exhibited the highest binding to rCRD4-MR (**Fig. S2A**), all three anti-CRD4-MR scFvs bound equally well to CD206^high^ macrophages. Anti-CRD4-MR scFv binding intensity was proportional to the levels of CD206 expressed by the macrophages and none of the anti-CRD4-MR scFvs bound to CD206^-^ monocytes (**Fig. S2B**). Finally, the pre-incubation of macrophages with rCRD4-MR protein blocked anti-CRD4-MR #G11 and #B2 scFv binding, further confirming the specificity of these scFvs for CD206 (data not shown).

**Figure 4:**
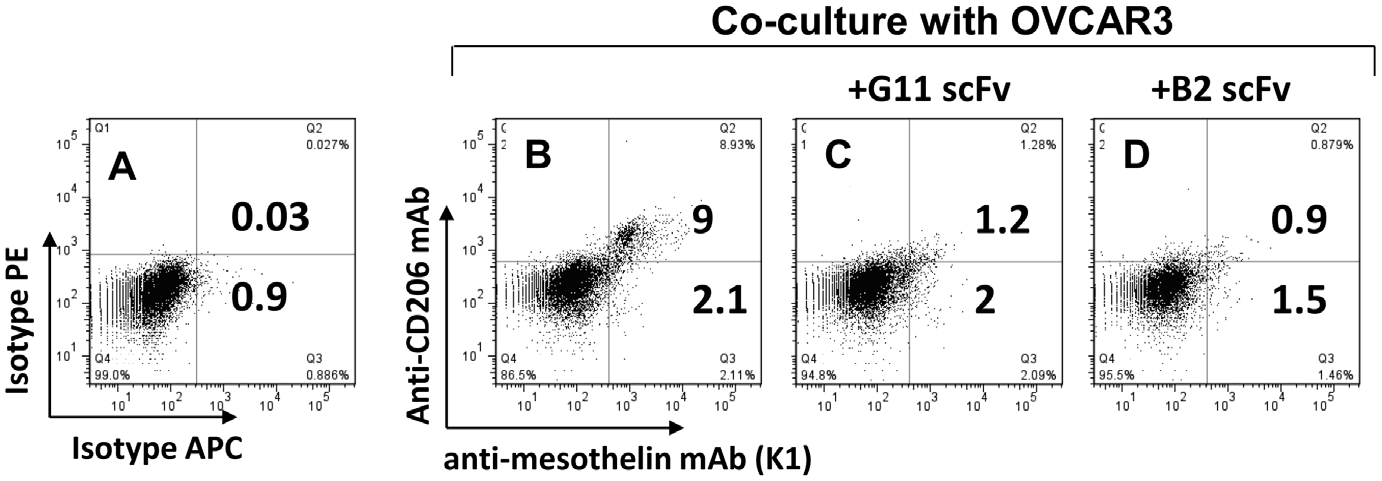
Blocking of mesothelin binding to CD206^low^ macrophages with anti-CRD4 MR scFvs. Macrophages were double stained with PE anti-CD206 mAb and APC anti-mesothelin antibody (K1) after *in vitro* differentiation with IFN-γ/LPS and 72 hr co-culture with OVCAR3 cells (**B**). Blocking conditions included anti-CRD4 MR scFvs #G11 (**C**) and #B2 (**D**). As negative control, CD206^low^ macrophages were labeled with isotype control antibodies (**A**). Results representative of 3 or more independent experiments.

We next tested the ability of anti-CRD4-MR scFvs to block mesothelin binding to macrophages during co-culture with tumor cells. **Figure 3B** shows that anti-CRD4-MR scFv #B2 could completely block tumor-cell released mesothelin binding to CD206^high^ macrophages, while the blocking activities of anti-CRD4-MR scFvs #G11 and #H11 were intermediate or low, respectively (**Fig. 3C,D** **)**. Anti-CRD4-MR scFv #G11 and #B2 could also block mesothelin binding to CD206^low^ macrophages co-cultured with tumor cells (**Fig. 4B–D**). These results further supported that tumor-released mesothelin binding to monocytes and macrophages was mediated by CD206.

### Anti-CDR4-MR scFv #G11 prevents tumor-induced polarization of CD206^low^ macrophages

**Figure 5:**
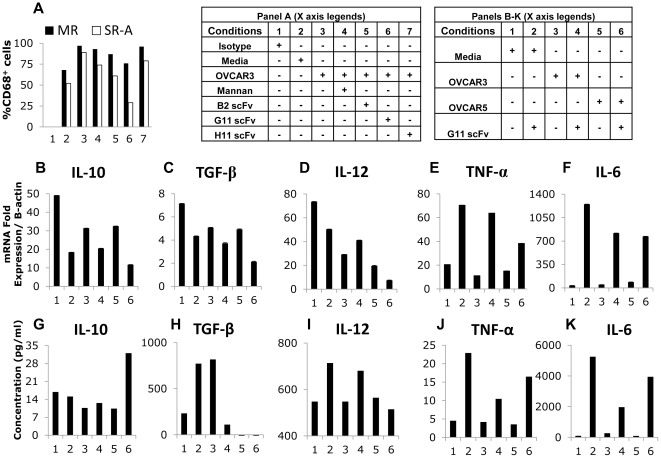
Effects of anti-CRD4 MR scFvs on the cytokine production of CD206^low^ macrophages during co-culture with tumor cells. **A**. Flow cytometry analysis of CD206 (**black bars**) and SR-A (**white bars**) expression of CD206^low^ macrophages after 72 hr-incubation in medium (**2**), or co-culture with OVCAR3 cells in medium (**3**), or in the presence of mannan (**4**), anti-CRD4 MR scFv #B2 (**5**), #G11 (**6**), or #H11 (7). As negative controls, macrophages were labeled with isotype control antibodies (**1**). **B–I**. CD206^low^ macrophages were incubated in medium (**1–2**), or co-cultured with OVCAR3 (**3–4**) or OVCAR5 (**5–6**) cells during 72 hrs. Five µg/ml of anti-CRD4 MR scFv #G11 were added in conditions **2**, **4 and 6**. Real-time PCR (**B–F**) and cytokine bead array analysis (**G–K**) were performed to measure (**B,G**) IL10; (**C,H**) TGF-β; (**D,I**) IL-12; (**E,J**) TNF-α and (**F,K**) IL-6. Results representative of 2 or more independent experiments.

**Figure 6:**
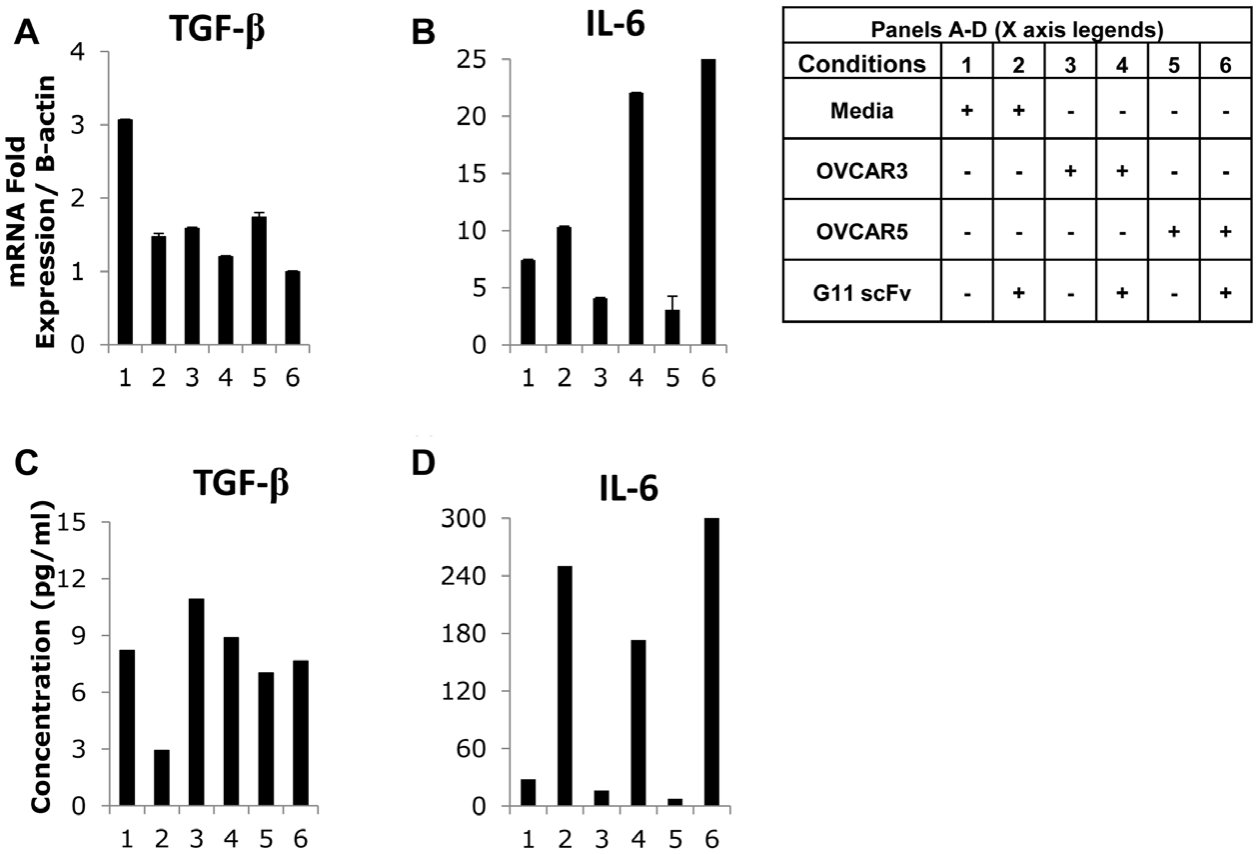
Effects of anti-CRD4 MR scFvs on CD206^high^ macrophage phenotype during co-culture with ovarian cancer cell lines. CD206^high^ macrophages were incubated in medium (**1–2**), or co-cultured with OVCAR3 (**3–4**) or OVCAR5 (5–6) cells during 72 hrs. Five µg/ml of anti-CRD4 MR scFv #G11 were added in conditions **2**, **4 and 6**. Real-time PCR (**B–C**) and cytokine bead array analysis (**D–E**) were performed to measure (**A,C**) TGF-β; and (**B,D**) IL-6. Results representative of 2 or more independent experiments.

Hagemann and colleagues demonstrated that ovarian tumor cells cause dynamic changes in the macrophage secretion profile of cytokines, chemokines and matrix metalloproteases [11]. We confirmed that our transwell co-culture settings could also induce tumor-induced polarization of monocytes and macrophages with upregulation of CD206 (**Fig. S3A**) and characteristic changes of cytokine profiles (**Fig. S3B,C**). To assess whether CD206 engagement could alter macrophage polarization, CD206^low^ and CD206^high^ macrophages were co-cultured with tumor cells in medium or in the presence of 1 mg/ml mannan or of 5 µg/ml of anti-CRD4-MR scFvs#B2, #H11, or #G11. After 3 days of co-culture, CD68+ macrophages were isolated and the expression of CD206 and scavenger receptor (SR-A) was analyzed by flow cytometry. Addition of anti-CRD4-MR scFv #G11 (**Fig. 5A** **, condition 6**) and, to a lower extend, of scFv #B2 (**Fig. 5A** **, condition 5**), could inhibit the upregulation of CD206 and SR-A in CD206^low^ macrophages co-cultured with tumor cells. The addition of mannan (**Fig. 5A** **, condition 4**) or of anti-CRD4-MR scFvs #H11 (**Fig. 5A** **, condition 7**) did not. We further analyzed the effects of anti-CRD4-MR scFvs #G11 on macrophage expression profiles for IL-10, TGF-β, IL-12, IL-6 and TNF-α at transcriptional (**Fig. 5B–F**) and protein levels (**Fig. 5G–K**). Anti-CRD4 MR scFv #G11 preserved CD206^low^ macrophage phenotype during co-culture with tumor cells, as shown by the up-regulation of IL-12 (**Fig. 5D,I** **, condition 4**), TNF-α (**Fig. 5E,J** **, conditions 4,6**), and IL-6 (**Fig. 5F,K** **, conditions 4,6)**. Consistent with these findings, IL-10 and TGF-β mRNA transcript levels (**Fig. 5B,C** **conditions 4,6**) were downregulated by the treatment with anti-CRD4-MR scFv #G11, as well as the TGF-β protein levels (**Fig. 5H** **, conditions 4,6**). Of note, IL-10 protein levels did not correlate with mRNA levels within the timeframe of our analysis (**Fig. 5G**). Anti-CRD4 MR scFv #G11 could also partially revert the CD206^high^ phenotype to that of a CD206^low^ for TGF-β and IL-6 production (**Fig. 6A–D** **, conditions 4,6**), but did not significantly affect the other analyzed cytokines (**Fig. S4**). These results suggest that anti-CRD4 MR scFv #G11 can control tumor-induced polarization of macrophages. Finally, because the effects of anti-CRD4 MR scFv #G11 on macrophage phenotype were more pronounced in the absence of tumor cells (**Fig. 5B–K** **, condition 2),** CD206 engagement by soluble mesothelin may compete with anti-CRD4-MR scFv binding.

### GPI anchor remains attached to soluble mesothelin after release by tumor cells

**Figure 7:**
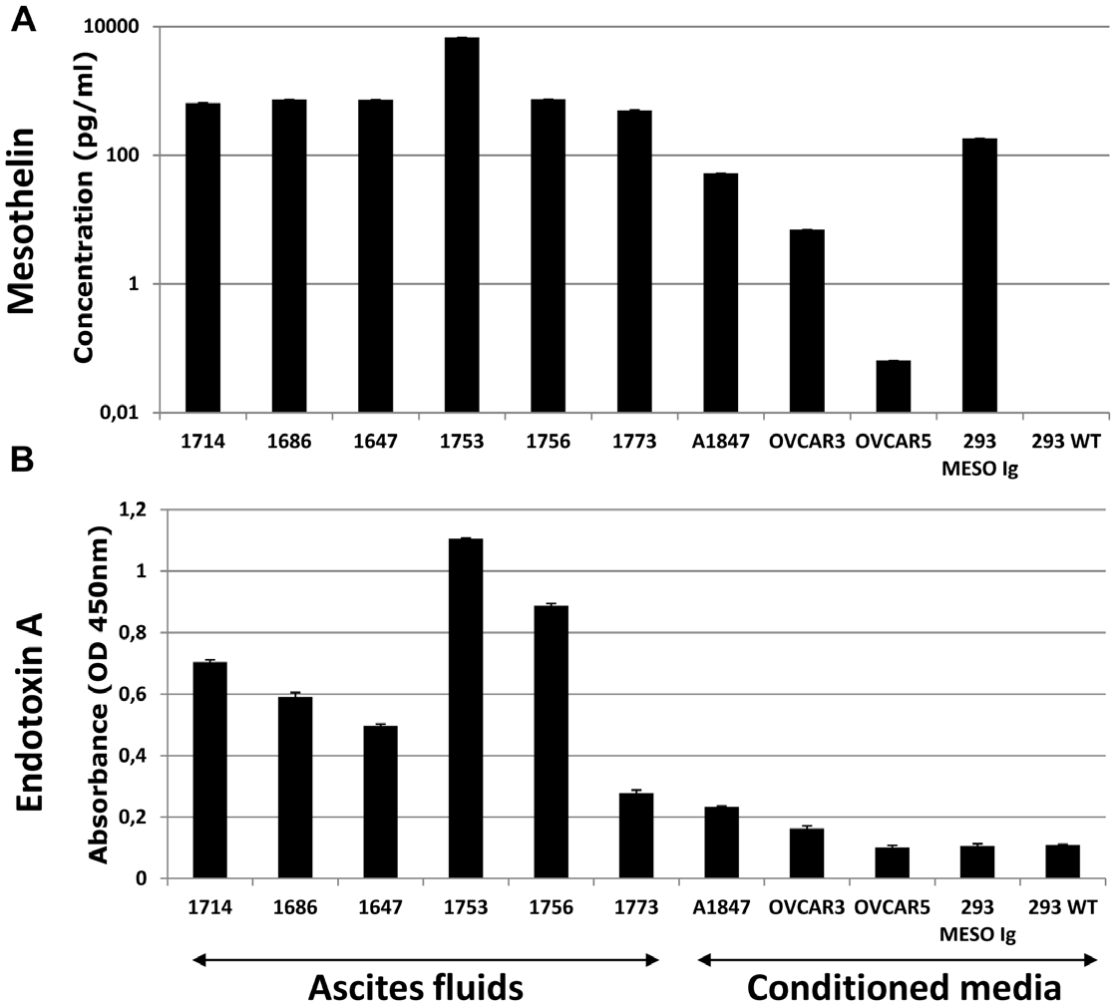
Detection of mesothelin in ovarian cancer ascites and conditioned media. ELISA assays of ascites fluids from ovarian cancer patients (#1714; #1686; #1647; #1753; #1756; #1773) and conditioning media derived either from ovarian cancer cell lines (A1847; OVCAR3; OVCAR5) or from 293 cell lines, wild type (293WT) or transfected to secrete a GPI anchor-truncated mesothelin fused to Ig (293 MESOIg). (**A**) Anti-mesothelin ELISA double determinant assay using anti-mesothelin mAbs as capture and detection antibodies (R&D Systems). Results representative of two independent experiments. (**B**) ELISA double determinant assay using anti-mesothelin mAb (K1) as capture antibody and biotinylated Endotoxin Alpha as detection reagent, followed by HRP-labeled streptavidin. Colorimetric signal was developed with TMB substrate solution, quenched with sulfuric acid and read at 450 nm on a Biotek ELISA reader.

Cell surface attachment of mesothelin depends on a glycophosphatidylinositol (GPI) anchor but, to our knowledge, the release mechanism of mesothelin from tumor cells has not been described. It was thus unclear whether soluble mesothelin remains linked to the GPI anchor in patient fluids or in tumor-conditioned media. To answer this question, we exploited two biochemical characteristics of GPI anchors that are 1/ GPI core specific composition in glycan moieties [69], and 2/ GPI anchor insertion in lipid raft microdomains [70], [71]. To address whether GPI anchors were attached to soluble mesothelin, we developed an ELISA double determinant assay using an anti-mesothelin antibody as capture reagent and Endotoxin alpha (Endo-A) as detection reagent; endo-A specifically binds to GPI core glycan moieties [72], [73], [74]. The assay is referred to as “Endo-A meso ELISA” in the rest of the study. We then compared the results of the Endo-A meso ELISA with these of a classical anti-mesothelin ELISA assay. **Figure 7** shows that, as expected, the anti-mesothelin ELISA assay detected soluble mesothelin in all ascites fluids as well as in conditioned media from ovarian cancer cell lines and from 293 MESOIg (a transformed cell line that expresses a GPI anchor-truncated mesothelin fused to an Ig domain [57]) (**Fig. 7A**). Endo-A meso ELISA also detected soluble mesothelin in ascites fluids and conditioned media from cancer cell lines. However, Endo-A meso ELISA could not detect soluble mesothelin released by 293 MESOIg cell line that is truncated for the GPI anchor (**Fig. 7B**). These results support the hypothesis that soluble mesothelin carries a GPI-anchor after tumor-release in ascites fluids and in tumor-conditioned media.

**Figure 8:**
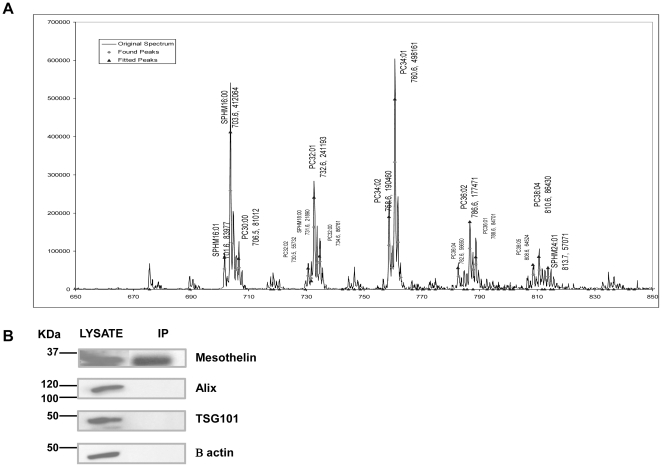
Lipid analysis of mesothelin. (**A**) Tandem mass spectrometry (ESI_MS2) of choline-containing phospholipids, phosphatidylcholine (PC) and sphingomyelin (SM). Lipids were extracted by the chloroform/methanol solvent Folch mixture from their association with the immunoprecipitated mesothelin from OVCAR3 cell culture supernatant. The high proportion of SM relatively to PC in the supernatant is revealed by the amplitude of peak m/z 703 (palmityl-SM) compared with peaks at m/z 732 (32∶1 PC), 760 (34∶1 PC) and 786 (36∶2 PC). Parent phospholipids of the phosphorylcholine ion (+184) are indicated as (total carbon number in acyl chains:double-bond number PC). Cholesterol assay by MS2 is obtained after acetylation of the non esterified sterol by recording of the transition 446 (acetyl-cholesterol + NH+4) to 369 (not shown). SM/cholesterol molar ratios are given as mole/mole after calibration. (**B**) Western Blot analysis of immunoprecipitated mesothelin from tumor conditioned media (right lane) and tumor cell lysates (left lane) as positive control. Membranes were probed with anti-Mesothelin, -TSG101, - Alix, or -β actin, as indicated. Signals were detected by ECL.

We next addressed whether mesothelin was inserted into lipid rafts. Mesothelin could be detected by an anti-mesothelin antibody (K1) in the lipid raft fractions extracted from OVCAR3 membranes and separated by electrophoresis, consistent with the fact that GPI-anchored proteins are associated with lipid raft domains (data not shown) [42], [43]. We then used tandem mass spectrometry to analyze the composition of lipids associated with soluble mesothelin. **Figure 8A** shows that soluble mesothelin immunoprecipitated from OVCAR3 conditioned medium is associated with lipids exhibiting a CHOL/SM ratio of 1 and a remarkably high SM/PC ratio (8.1). The most abundant molecular species of SM is comprised of the saturated palmityl-SM (m/z 703). These ratios of CHOL and saturated SM were consistent with lipid raft composition. These results support the fact that tumor-released mesothelin remains associated lipid remnants of rafts.

Lastly, we investigated whether mesothelin resides in tumor-released exosomes, We immunoprecipitated mesothelin from tumor cell supernatants and looked by western blot for the presence of co-immunoprecipitated exosomal proteins TSG101 and ALIX [75]. Tumor cell lysate (**Fig. 8B** **, left lane**) was used as a positive control. TSG101 and ALIX exosomal proteins were detected only in OVCAR3 cell lysate (**Fig. 8B** **, right lane**). We conclude that the absence of co-immunoprecipitation of exosomal proteins with tumor-released mesothelin demonstrates that mesothelin does not reside in tumor-released exosomes.

## Discussion

Polarized inflammation is a hallmark of several pathologic conditions including infection and cancer, and plays a central role in disease progression and/or resolution. Tumor associated macrophages (TAMs) are critical for cancer growth and development, but the signals eliciting TAM phenotype remain incompletely understood. We provide here the first evidence that the soluble cancer biomarker mesothelin binds to macrophages. To analyze the functional consequences of the binding, we isolated human recombinant antibodies directed against the domain 4 of the mannose receptor (anti-CRD4-MR scFv). Anti-CRD4-MR scFv #G11 could block the binding of tumor-released mesothelin to mannose receptor and prevent *in vitro* tumor-induced TAM polarization. We further demonstrated that the interaction of tumor-released mesothelin with mannose receptor expressed by macrophages was mediated, at least in part, by mesothelin GPI anchor. The attachment of tumor-released mesothelin to a GPI anchor was shown using a novel ELISA assay that detects soluble molecules bearing both a mesothelin epitope and GPI core glycan moieties, as well as by lipid profiling of mesothelin immunoprecipitated from tumor cell conditioned media. No co-immunoprecipitation of exosomal proteins with mesothelin was observed, excluding the possibility that other surface proteins released in exosomes could mediate mesothelin interaction with mannose receptor. These results support the hypothesis that tumor-released mesothelin GPI anchor contributes to TAM polarization through the engagement of mannose receptor.

Tumor overexpression of glycoproteins such as mesothelin has mainly been used as a mean of biomarker identification [76], [77], [78], [79], [80], [81], but the study of their functional roles during cancer development remains preliminary [82], [83]. Patankar and colleagues discovered that CA125, the most studied biomarker for ovarian cancer, binds to NK cells via siglec-9, and that the binding suppresses NK function [84], [85], [86]. Allavena and colleagues recently described the active role of mucin proteins such as TAG-72 and CA125 in promoting an immune suppressive phenotype of human TAMs [87]. Our results suggest that mesothelin GPI anchor contributes to macrophage phenotype polarization. Altogether, these findings highlight the ability of tumor antigens to suppress tumor rejection through the manipulation of innate immunity.

Pattern recognition receptors (PRR) are central to innate immunity and include toll-like receptors (TLRs) and mannose receptor (CD206/MR). MR binding by exogenous and endogenous factors has been reported to elicit diverse cell stimulation and differentiation programmes in a ligand-dependent manner. For example pathogen binding to MR triggers NF-κB activation [88] or PPARgamma activation [89], [90]. In addition, recent studies indicate that MR complements TLR signalling in proinflammatory responses [91] and specifically synergizes with TLR2 in activating a NF-κB-dependent proinflammatory responses [92]. TLR2 recognizes lipoproteins and peptidoglycans [93] from exogenous origins such as protozoa GPI anchors [94], as well as from endogenous origin such as versican, an extracellular matrix proteoglycan upregulated in many human tumors and a potent inducer of macrophage activation [95]. The ability of cancer cells to subvert components of the host innate immune system and promote an inflammatory microenvironment favorable for cancer growth, including soluble factors that bind to myeloid cells [95], [96], [97], makes it conceivable that mannose receptor engagement by GPI anchors linked to tumor antigens such as mesothelin, folate receptor, CEA, and CaMOV18 [98], may represent another cancer strategy to escape immune surveillance [99]. A better understanding of the molecular mechanisms underlying PRR engagement by tumor antigens may lead to substantial new insights with important implications for the development of novel therapeutics for cancer treatment. We conclude that anti-CRD4-MR scFv #G11 can prevent tumor-induced macrophage dynamic changes, which provides a proof of principle for the targeting CRD4-MR mannose binding domain as a mean to re-set the innate immune response towards tumor rejection. We propose that interfering with tumor antigen binding to MR could prevent TAM polarization and have therapeutic potential against solid tumors.

## Materials and Methods

### Human samples

Healthy Monocytes were obtained from the Human Immunology Core of the University of Pennsylvania. Ascites and solid tumors samples from ovarian cancer patients were obtained from the Ovarian Cancer Research Center's patient sample repository of the University of Pennsylvania.

### Antibodies

Anti-mesothelin ELISA kit (catalog # DY3265) was purchased from R&D Systems. Anti-human CD206-PE, CD206-APC, CD163-PE mouse monoclonal antibodies (mAb) and 7-AAD were obtained from BD. Anti-human, anti-mouse CD68-FITC and anti-V5 mAb-AF647 were from Serotec. Anti-human Mesothelin (K1), K1-PE, anti c-myc mAb and anti-mouse HRP labeled antibody were purchased from Santa Cruz. APC, PE-labeled or HRP-labeled streptavidin (SA-APC, SA-PE and SA-HRP, respectively), anti-human IL-10-PE, IL-12-PE, CD14-FITC, CD14-PE-CY7, CD45-APC-CY5 mAbs, and rat anti-mouse IL-10-APC, IL-12-PE, CD11b-PerCpCy5, CD45-APC-Cy7, Brefeldin A were purchased from eBiosciences. SA-polyHRP was purchased from Fitzerald (PolyHRP80 Streptavidin, catalog #65R-S105PHRP). The isotype controls mIgG-PE, rIgG-PE, rIgG-APC, rIgG-PerCpCy5, rIgG-APC-Cy7, and rIgG-PE-Cy7 were from eBiosciences and anti-mouse-AF488 from Invitrogen. Goat anti-human mesothelin was purchased from R&D Systems (cat# AF3265). Mouse anti-human Alix was purchased from AbD Serotec. Rabbit anti-human TSG101 and goat anti-rabbit HRP were from Abcam. Anti-human B-actin conjugated to HRP was obtained from Sigma.

### Cell culture

#### Cell lines

Ovarian cancer cell lines Ovcar3, Ovcar5, A1847 and C30, as well as wild type (WT) 293, were acquired from ATCC. 293 MESOIg was derived from 293 WT, as described in [63].

#### *In vitro* maturation of monocytes

The procedure of macrophages differentiation was adapted from Porcheray et al [7]. Briefly, freshly purified monocytes from healthy donors (Human Immunology Core of the University of Pennsylvania) were cultured at a density of 1×10^6^ /ml in the presence of 10 ng/ml of M-CSF and 1 ng/ml of GM-CSF for 8 days. Media with growth factors was refreshed at day 3 and at day 6. Further polarization was induced with 10 ng/ml of IFN-γ and LPS for 4 days to obtained CD206^low^ macrophages or with 10 ng/ml IL4 and IL-10 to obtain CD206^high^ macrophages (**Fig. S1**). In some experiments, monocytes were incubated 3 days in the presence of M-CSF and GM-CSF and incubated with tumor cells in transwells to upregulate CD206 expression.

#### Transwell co-culture

Ovarian cancer cell lines OVCAR3 and OVCAR5, 293 MESOIg or WT 293 cell lines were collected using versene and plated at 0.5×10^6^ on the bottom part of 6-well transwell plates. Freshly isolated monocytes or *in vitro* maturated macrophages were collected by gentle scrapping and plated at 1×10^6^ in the transwell inserts. Cells were at first co-cultured in RPMI supplemented with 10% FBS at 37°C in presence of 5% C02. CD206^null^ monocytes and CD206^low^ macrophages co-cultured for 3 days with OVCAR3 cells consistently upregulated CD206 expression and developed an alternative phenotype (**Fig. S3**). For blocking assays, mannan (1 mg/ml, Sigma) or scFvs (5 µg/ml) were added to culture medium at day 1 and renewed at day 2. In other experiments, blocking of the non-specific binding of mouse monoclonal antibodies to macrophage Fc receptors was obtained using serial dilutions of human IgG protein at concentrations of 0.01 mg/ml, 0.1 mg/ml or 1 mg/ml, added at day 0 and day 2 to. At day 3, macrophages were collected from the transwell inserts by pipetting and directly used for flow cytometry staining or RNA extraction.

### Isolation of anti-CRD4-MR recombinant antibodies (scFv)

Mannoses specifically bind to the domain 4 of mannose receptor (CRD4-MR) (gi#145312260) [100]. To isolate scFv capable to block mannose binding to the mannose receptor, we first cloned the cDNA encoding CRD4-MR from CD14^+^ cells isolated from an ovarian cancer patient ascites. First strand cDNA was synthesized using 1 µg of RNA from ascites CD14^+^ cells by reverse transcription PCR using oligo-dT primers. cDNA encoding CRD4-MR was amplified by PCR using the forward primer 5′-ggtggaggttctggtggtggtggatctgatgttttgaaatgtgatgaaaaggc-3′, and the reverse primer 3′-ctactatgtcttggaatgatattaatgtcgacggtaagcctatccctaaccctctcctcggtc-′5) that enabled the addition of recombination sequences for cloning by gap repair in p416 BCCP vector for yeast-secretion [66]. CDR4-MR cDNA PCR fragment was then purified by gel extraction (Qiagen gel extraction kit), verified by sequencing for identity with the published sequence and inserted by gap repair in p416 BCCP vector. Yeast secreted CRD4-MR recombinant protein was validated by western blot using anti-V5-HRP mAb. The isolation of anti-CRD4-MR scFv from a yeast-display scFv library was performed as described in [66] with the following modifications. The yeast-display scFv library was first enriched by two rounds of magnetic sorting using 45.5 pmol of CRD4-MR recombinant protein (rprot) biotinylated with EZ-link Sulfo-NHS-LC-Biotin kit (Pierce). Screenings were then performed by three rounds of flow sortings using BD FACSAria™ cell sorter and 22.72 pmol to 2.27 pmol of CRD4-MR rprot. CRD4-MR-specific yeast-display sub-library was shuffled into p416 BCCP yeast-secreting vector.

The first validation of scFv binding to CRD4-MR was performed by capture ELISA as described in [68]. Amino plates with coated with serial dilutions of His-purified scFv diluted from 10 to 0.001 µg/ml in carbonate-bicarbonate buffer (Sigma). Biotinylated CRD4-MR was added at 0.2 µg/ml and detected by SA-HRP and TMB (KPL). ELISA plates were read as described for ELISA double determinant assays. Anti-CRD4 scFvs were then tested by flow cytometry for their binding to CD206^low^ and CD206^high^ macrophages. Anti-CRD4-MR scFvs (5-10 µg/ml) were first preincubated with AF647-labeled anti-V5 mAb (1 to 2.5 µg/ml). Anti-V5/CRD4-MR scFvs were then incubated with macrophages for 30 min at 4°C in FACS buffer (PBS/2%FBS) and the binding was analyzed on BD FACScanto I instrument.

### Cell characterization

#### Macrophage phenotyping by RT PCR

RNA from 0.6×10^6^ macrophages was isolated using Trizol as recommended by the manufacturer (Invitrogen). cDNA was amplified from 1 µg of RNA by random priming and real time PCR was performed in triplicates using Applied Biosystem's primers for IL-12-p35, IL-10, TNF-α, IL-6, β-actin. Data acquisition and analysis was performed according Applied Biosystem's instructions.

#### Flow cytometry analysis

Prior to staining, non specific binding sites on macrophages were blocked by incubation with mouse IgG or CD16/32 Ab (5 µg/ml) for 10 min at 4°C. Abs were added at manufacturer recommended concentrations and incubated for 30–45 min at 4°C in the dark. 7-AAD was added to distinguish dead cells 15 min before data acquisition. Detection of mesothelin on macrophage surface was performed in a buffer containing calcium (10 mM Hepes, 140 mM NaCl, 2.5 mM CaCl2, 1%FBS, 0.1% NaN3) to maintain the lectin binding properties of CD206 [101] for 30 min at 4°C in the dark. **Sodium azide was used to prevent passive endocytosis. Intracellular staining was** performed after surface labeling; cells were incubated in permeabilization/fixation buffer (eBiosciences) and incubated with antibodies for intracellular staining as recommended by the manufacturer. Data were obtained with BD FACScanto I instrument.

#### Cytokine Bead Array

Cytokine quantification was performed using the multiplex kit for human IL-12-p70, IL-10, IL-6, IFN-γ, TNF-αand monoplex kit for human TGF-β from BD Biosciences. Assays were performed using manufacturer's instructions.

### Double determinant ELISA assays for detection of soluble mesothelin and of GPI-anchor mesothelin

Detection of soluble mesothelin was performed using the Human Mesothelin DuoSet kit (R&D Systems), as recommended by the manufacturer. Ascites supernatants were diluted 1/100 and 1/1000 and cell line conditioned media was used undiluted or 1/10 diluted in diluent (PBS/1% BSA). Detection of GPI-anchor mesothelin was performed using anti-mesothelin K1 mAb at 5 ug/ml as capture reagent and biotinylated-Endotoxin A at 2 ug/ml as detection reagent, followed by SA-polyHRP and TMB (KPL). Ascites supernatants were used undiluted or 1/100 diluted in diluent (PBS/1% BSA); cell line conditioned media were used undiluted. Plates were read using an ELISA plate reader (Biotek) at 450 nm.

### Biotinylated-Alpha Toxin

The plasmid pBRS10 encoding native alpha toxin [74] expressing a histidine-tag was transformed BL21(DE3) pLysS E. coli. Bacteria were grown in 2XYT media at 37°C overnight. The culture was diluted 20 fold, at 1.0 OD protein expression was induced for 4 hours at room temperature using 0.2 mM IPTG. Bacterial pellets were resuspended in 0.5X PBS with protease inhibitors and lysed using a French Press. Alpha toxin was purified from the supernatant using Talon cobalt resin (Clontech). Bound toxin was eluted using step immidazole gradients in 25 mM MES pH 6.5 buffer supplemented with 150 mM NaCl. Alpha toxin fractions were pooled and dialyzed to remove immidazole prior to SP cation-exchange chromatography. Alpha toxin fractions were concentrated and buffer exchanged into 25 mM MES pH 6.5, 150 mM NaCl before storage at -80°C. Purified toxin was dialyzed into 1X PBS pH 9.0 prior to biotin labeling using Sulfo-NHS-LC-Biotin (Pierce) as recommended by the manufacturer followed by buffer exchange using a 10,000 MWCO membrane.

### Lipidomic analysis

Isolation of lipid raft fraction from OvCar3 whole membrane was performed after cell lysis by three rounds of freeze-thawing in presence of 20 mM Tris-HCl pH 7.5, 0.5 mM EDTA and protease inhibitor cocktail. Cell homogenates were passed through a 26-gauge needle 10 times, sonicated (3 pulses of 15 sec., on ice), and separated by sucrose gradient (40-5%). After ultracentrifugation at 200,000 g for 14 hrs, lipid rafts appeared as two discrete bands that were separated by electrophoresis and probed by western blot with anti-mesothelin mAb K1 at dilution 1/1000 (data not shown).

Mesothelin was immunoprecipitated from 50 ml of OVCAR3 conditioned medium (CM). CM was harvested, concentrated 10 times (Millipore centrifuge concentrator, 10 K cut-off), and incubated overnight with 5 µg/ml of anti-mesothelin biobody P4 [63] at 4°C. Mesothelin/P4 complexes were retrieved with 150 µl of pre-washed Dynal Myone streptavidin magnetic beads (Myltenyi), for 2 hrs at 4°C. Beads bound to mesothelin/P4 complexes were magnetically separated, validated by western blot for the presence of bound mesothelin using anti-mesothelin mAbs and submitted to lipid analysis by tandem mass spectrometry.

### Tandem mass spectrometry

Lipid molecular profiles were obtained for each separated class (phosphatidylcholine (PC), sphingomyelin (SM), cholesterol acetyl ester) using the triple quadrupole API3000 (AB Sciex, Toronto, Canada). The parent molecular species of lipids varies as a function of their fatty acid composition. The scan of parent lipids is obtained as the precursor of a class specific product ion cleaved after low energy collision induced dissociation (CID) such as phosphorylcholine (+184) for phosphatidylcholine and sphingomyelin or dehydrated cholesterol anion (+364) for sterides [45], [102].

### Western Blot analysis

Immunoprecipitated mesothelin from tumor conditioned media and tumor cell lysates were loaded on pre-cast gradient (4-15%) gels and allowed to run for 60 min, 120V. Protein were transferred by semidry transfer on Immobilon P transfer membrane for 30 min. Membranes were blocked overnight with 5% milk/PBST and blotted for Mesothelin, TSG101 and Alix, using goat anti-human mesothelin, rabbit anti-human TSG101, and mouse anti-human Alix, respectively, at 1 µg/ml. Membranes were washed 3 times with PBST and blotted with secondary antibodies to mouse, goat and rabbit at a dilution of 1/5,000. β-actin was detected using HRP-labeled anti-human β-actin at a dilution of 1/30,000. Membranes were incubated with ECL Plus (GE Healthcare) for 5 min and exposed to films for 15–30sec.

## Supporting Information

Figure S1 **Phenotype characterization of CD206^low^ and CD206^high^ macrophages. A-B**. Reverse microscopy analysis (**A**) and flow cytometry analysis (**B**) for extracellular expression of CD68, CD163 and CD206 (as indicated) of CD206^low^ (**upper panels**) and CD206^high^ macrophages (**lower panels**). **C–D.** mRNA levels and intracellular expressions of IL-12 (**upper panels**) and IL-10 (**lower panels**) at different time points after cytokine stimulation, as indicated.
(TIF)

Figure S2 **Validation of anti-CRD4 MR scFvs. A**. Capture ELISA. Serial dilutions (10-0.01 µg/ml) of plastic immobilized anti-CRD4 MR scFvs #G11 (**open squares**), #B2 (**open diamonds**) and #H11 (**open triangles**) were incubated with 0.2 µg/ml of biotinylated recombinant CRD4-MR protein (**lines**) or 2 µg/ml of irrelevant control antigen (**black square**). Binding was detected with SA-HRP. Colorimetric signal were developed with TMB substrate solution, quenched with sulfuric acid and read at 450 nm on a Biotek ELISA reader. **B**. Flow cytometry analysis. Anti-CRD4-MR scFvs #B2, #G11 and #H11 were premixed with anti-V5 mAb and incubated with (**upper panels**) CD206^null^ monocytes, (**middle panels**) CD206^low^ macrophages, and (**lower panels**) CD206^high^ macrophages. As positive controls, macrophages were labeled with anti-CD206 mAb (**left panels**). **Solid lines**, anti-mannose receptor antibodies or recombinant antibodies (scFv); **grey areas**: isotype control IgG1 mAb.
(TIF)

Figure S3 **Characterization of monocytes and CD206^low^ macrophages after co-culture with OVCAR3 ovarian cancer cell line.** **A**. Flow cytometry analysis of the percentage of CD206^null^ monocytes (**black bars**) and of CD206^low^ macrophages (**white bars**) that expressed CD206, before or after 72hr co-culture, as indicated. **B–C.** Transcriptional analysis of (**B**) CD206^null^ monocytes and (**C**) CD206^low^ macrophages for TGF-β, IL-10, IFN-γ, TNF-α, IL-12, IL-6 after 72hr-incubation in medium or co-culture, as indicated.
(TIF)

Figure S4 **Effects of mannan and anti-CRD4 MR scFvs on CD206^high^ macrophage phenotype during co-culture with ovarian cancer cell lines. A**. Flow cytometry analysis of CD206 (**black bars**) and SR-A (**white bars**) expressions on CD206^high^ macrophages incubated in medium or co-cultured with OVCAR3 cells in medium or in the presence of mannan, or anti-CRD4 MR scFv #B2, #G11 or #H11. As controls, macrophages were stained with isotype control antibodies. **B-I**. CD206^high^ macrophages were incubated in medium (**1–2**) or co-cultured with OVCAR3 (**3–4**) or OVCAR5 (**5–6**) cells for 72hrs. 5 µg/ml of anti-CRD4 MR scFv #G11 was added in the conditions **2, 4** and **6**. Real-Time PCR (**B–E**) and cytokine bead arrays (**F–I**) were performed to measure IL-10 (**B,F**), IL-12 (**C,G**), and TNF-α (**D,H**).
(TIF)

Table S1 **Germline immunoglobulin gene usage of the predicted amino-acid sequence of the anti-CRD4 MR scFvs B2, G11 and H11.** The homology of light (L) and heavy (H) chain variable regions to germline immunoglobulin genes is displayed for each anti-CRD4 MR scFv.
(TIF)
