# Supplementary figures and images for: Mannose Receptor (MR) Engagement by Mesothelin GPI Anchor Polarizes Tumor-Associated Macrophages and Is Blocked by Anti-MR Human Recombinant Antibody
Source: PLoS One. 2011 Dec 6;6(12):e28386. doi: 10.1371/journal.pone.0028386 (PMC3232216; doi:10.1371/journal.pone.0028386)

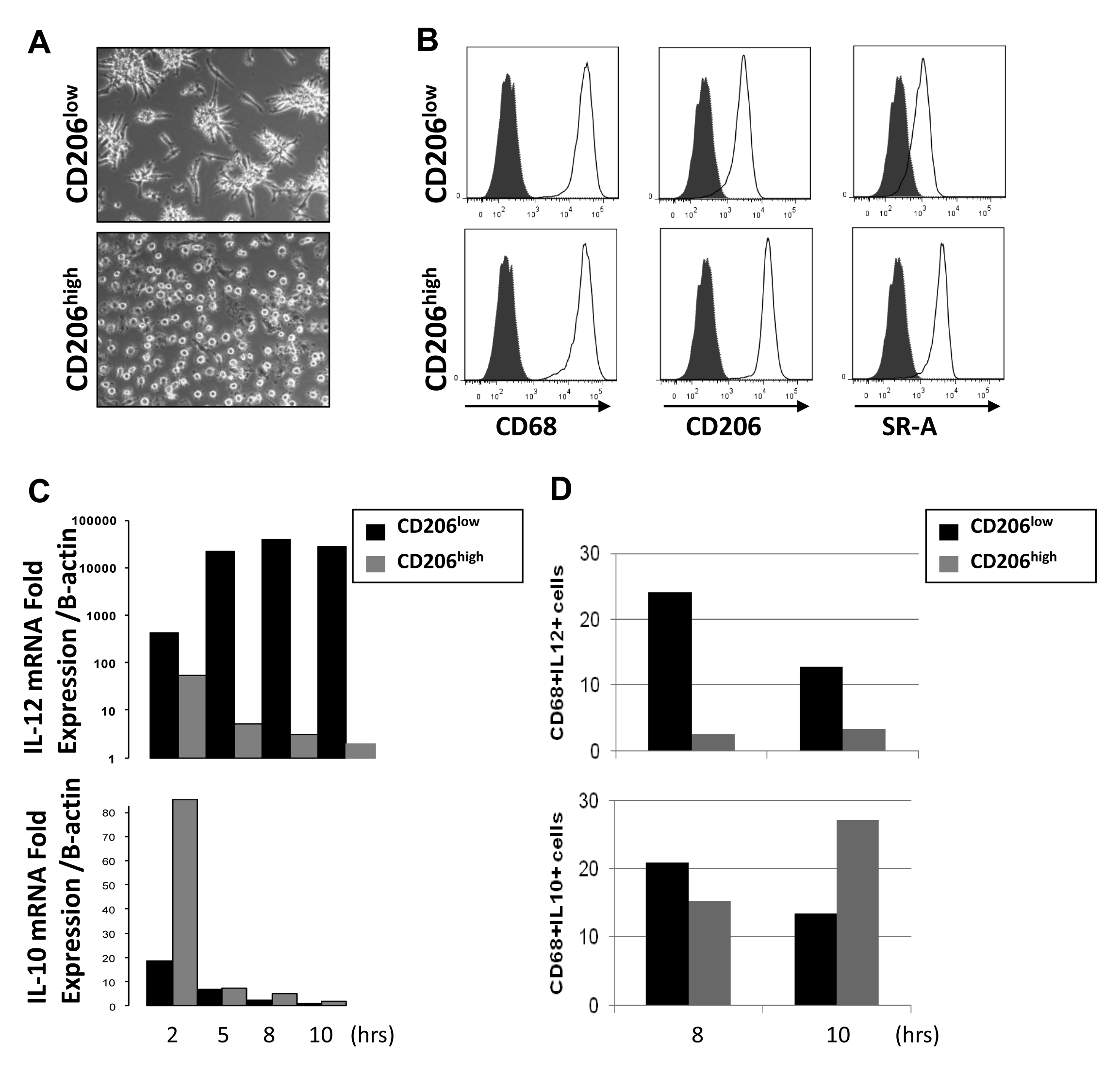

Supplement: Figure S1 — Phenotype characterization of CD206low and CD206high macrophages. A-B. Reverse microscopy analysis (A) and flow cytometry analysis (B) for extracellular expression of CD68, CD163 and CD206 (as indicated) of CD206low (upper panels) and CD206high macrophages (lower panels). C–D. mRNA levels and intracellular expressions of IL-12 (upper panels) and IL-10 (lower panels) at different time points after cytokine stimulation, as indicated. (TIF) [file pone.0028386.s001.tif]

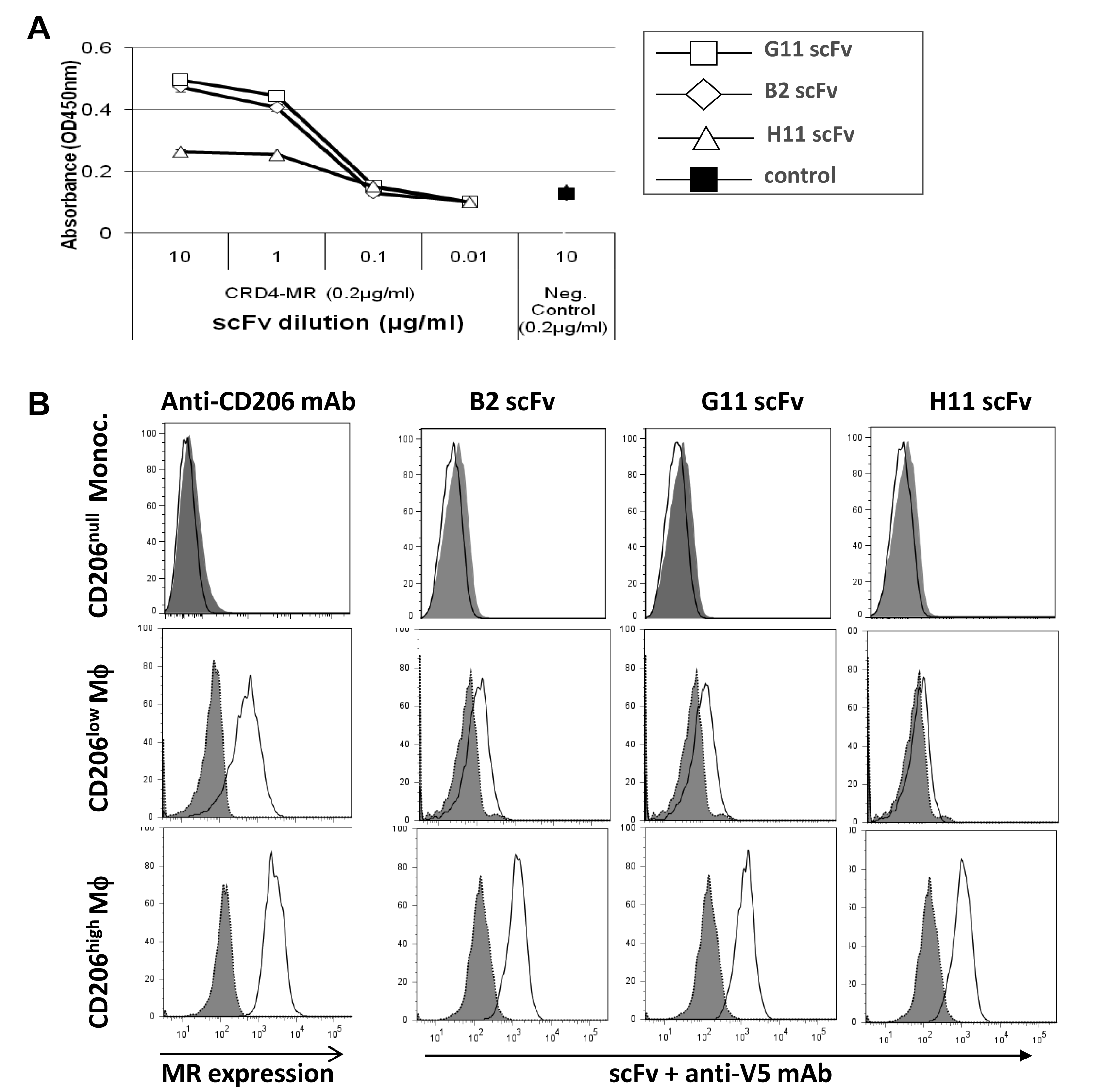

Supplement: Figure S2 — Validation of anti-CRD4 MR scFvs. A. Capture ELISA. Serial dilutions (10-0.01 µg/ml) of plastic immobilized anti-CRD4 MR scFvs #G11 (open squares), #B2 (open diamonds) and #H11 (open triangles) were incubated with 0.2 µg/ml of biotinylated recombinant CRD4-MR protein (lines) or 2 µg/ml of irrelevant control antigen (black square). Binding was detected with SA-HRP. Colorimetric signal were developed with TMB substrate solution, quenched with sulfuric acid and read at 450 nm on a Biotek ELISA reader. B. Flow cytometry analysis. Anti-CRD4-MR scFvs #B2, #G11 and #H11 were premixed with anti-V5 mAb and incubated with (upper panels) CD206null monocytes, (middle panels) CD206low macrophages, and (lower panels) CD206high macrophages. As positive controls, macrophages were labeled with anti-CD206 mAb (left panels). Solid lines, anti-mannose receptor antibodies or recombinant antibodies (scFv); grey areas: isotype control IgG1 mAb. (TIF) [file pone.0028386.s002.tif]

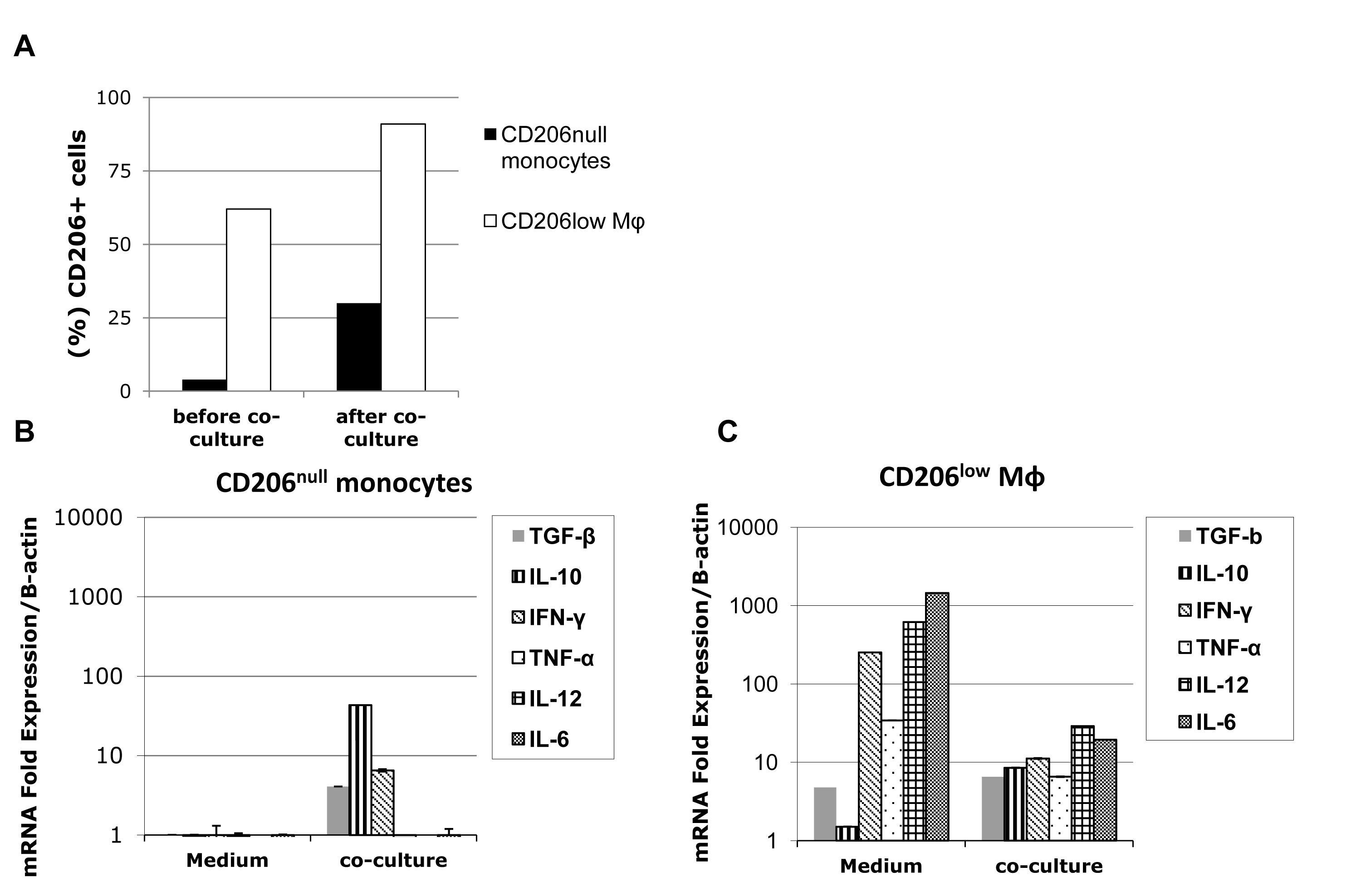

Supplement: Figure S3 — Characterization of monocytes and CD206low macrophages after co-culture with OVCAR3 ovarian cancer cell line. A. Flow cytometry analysis of the percentage of CD206null monocytes (black bars) and of CD206low macrophages (white bars) that expressed CD206, before or after 72hr co-culture, as indicated. B–C. Transcriptional analysis of (B) CD206null monocytes and (C) CD206low macrophages for TGF-β, IL-10, IFN-γ, TNF-α, IL-12, IL-6 after 72hr-incubation in medium or co-culture, as indicated. (TIF) [file pone.0028386.s003.tif]

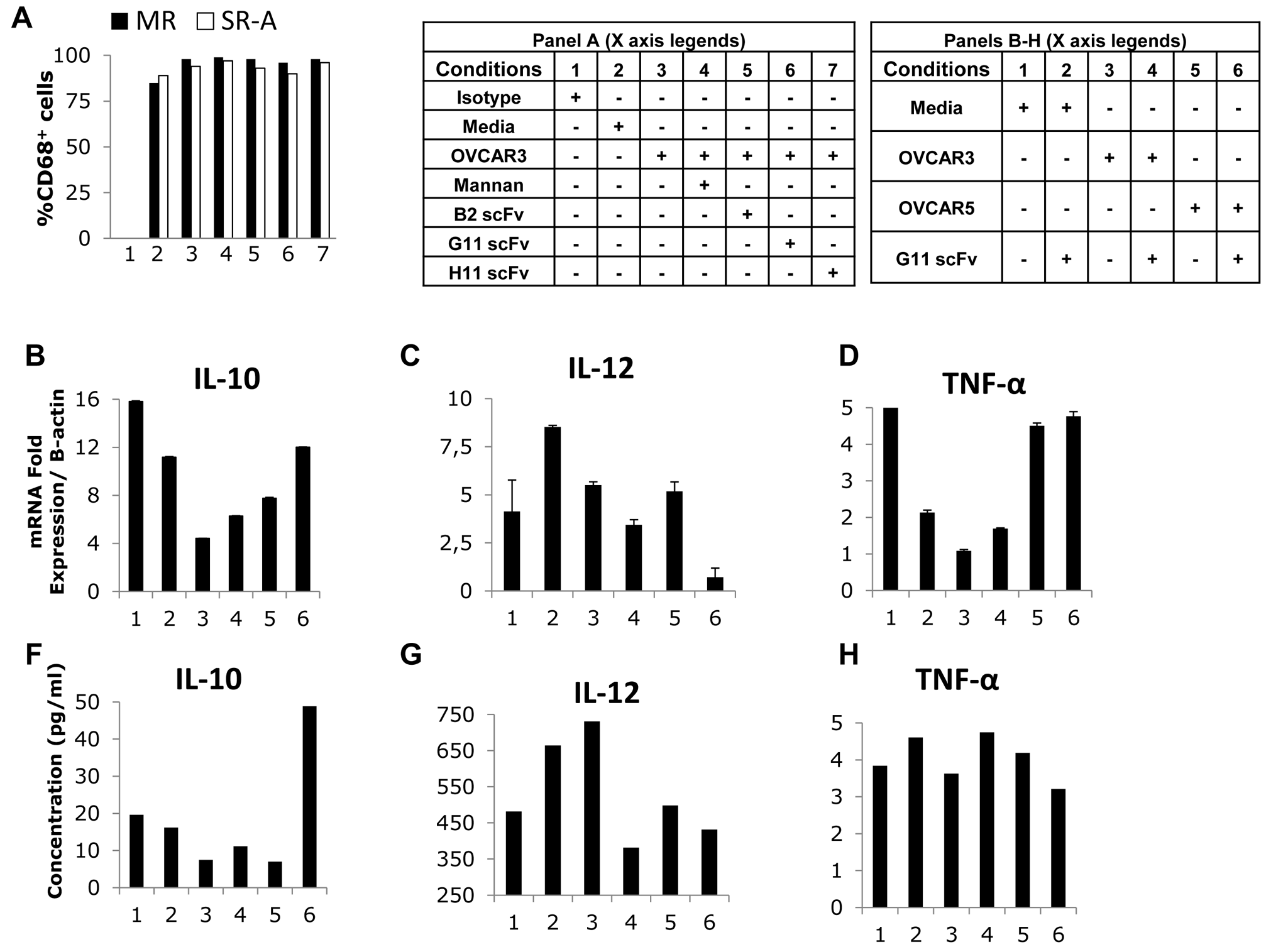

Supplement: Figure S4 — Effects of mannan and anti-CRD4 MR scFvs on CD206high macrophage phenotype during co-culture with ovarian cancer cell lines. A. Flow cytometry analysis of CD206 (black bars) and SR-A (white bars) expressions on CD206high macrophages incubated in medium or co-cultured with OVCAR3 cells in medium or in the presence of mannan, or anti-CRD4 MR scFv #B2, #G11 or #H11. As controls, macrophages were stained with isotype control antibodies. B-I. CD206high macrophages were incubated in medium (1–2) or co-cultured with OVCAR3 (3–4) or OVCAR5 (5–6) cells for 72hrs. 5 µg/ml of anti-CRD4 MR scFv #G11 was added in the conditions 2, 4 and 6. Real-Time PCR (B–E) and cytokine bead arrays (F–I) were performed to measure IL-10 (B,F), IL-12 (C,G), and TNF-α (D,H). (TIF) [file pone.0028386.s004.tif]

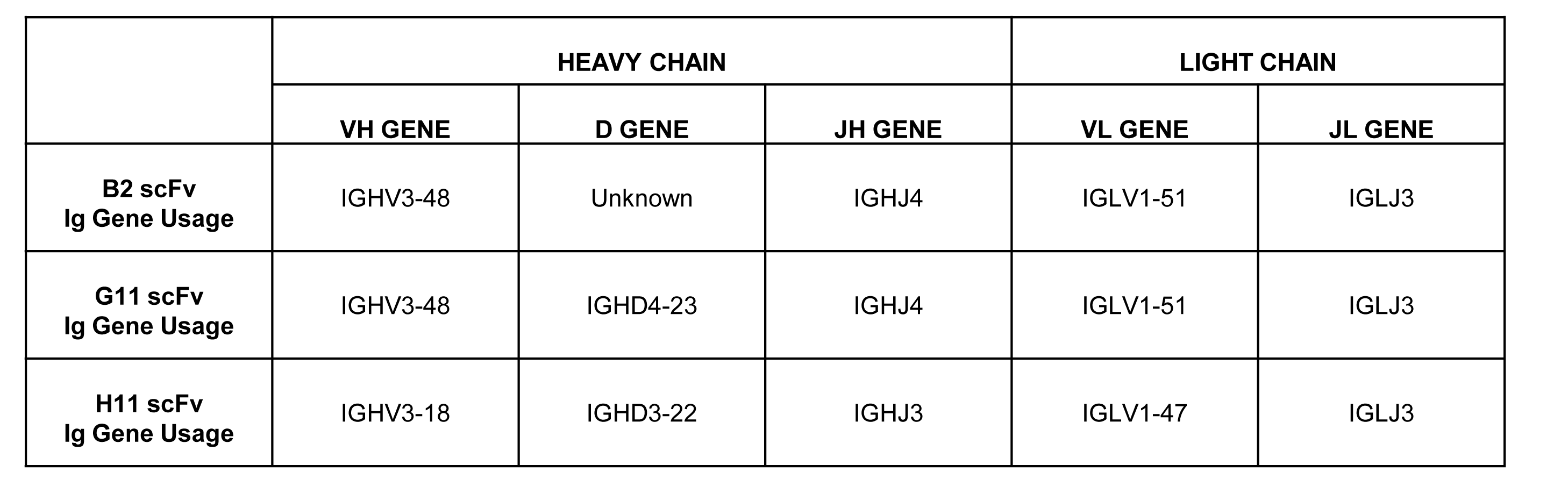

Supplement: Table S1 — Germline immunoglobulin gene usage of the predicted amino-acid sequence of the anti-CRD4 MR scFvs B2, G11 and H11. The homology of light (L) and heavy (H) chain variable regions to germline immunoglobulin genes is displayed for each anti-CRD4 MR scFv. (TIF) [file pone.0028386.s005.tif]
